# Transcriptome analysis of flowering regulation by sowing date in *Japonica* Rice (*Oryza sativa* L.)

**DOI:** 10.1038/s41598-021-94552-3

**Published:** 2021-07-22

**Authors:** Min Yin, Hengyu Ma, Mengjia Wang, Guang Chu, Yuanhui Liu, Chunmei Xu, Xiufu Zhang, Danying Wang, Song Chen

**Affiliations:** China National Rice Research Institute, Chinese Academy of Agricultural Sciences, Hangzhou, 310006 Zhejiang China

**Keywords:** Plant ecology, Plant physiology

## Abstract

Hybrid *japonica* cultivars, such as the Yongyou series, have shown high yield potential in the field in both the early and late growing seasons. Moreover, understanding the responses of rice flowering dates to temperature and light is critical for improving yield performance. However, few studies have analyzed flowering genes in high-yielding *japonica* cultivars. Based on the five sowing date experiments from 2019 to 2020, select the sensitive cultivar Yongyou 538 and the insensitive cultivar Ninggeng 4 and take their flag leaves and panicles for transcriptome analysis. The results showed that compared with sowing date 1 (6/16), after the sowing date was postponed (sowing date 5, 7/9), 4480 and 890 differentially expressed genes (DEGs) were detected in the leaves and panicles in Ninggeng 4, 9275 and 2475 DEGs were detected in the leaves and panicles in Yongyou 538, respectively. KEGG pathway analysis showed that both Ninggeng 4 and Yongyou 538 regulated rice flowering through the plant circadian rhythm and plant hormone signal transduction pathways. Gene expression analysis showed that *Os01g0566050* (*OsELF3-2*), *Os01g0182600* (*OsGI*), *Os11g0547000* (*OsFKF1*), *Os06g0275000* (*Hd1*), and *Os09g0513500* (*FT-1*) were expressed higher and *Os02g0771100* (*COP1-1*) was expressed lower in Yongyou 538 compared with Ninggeng 4 as the climate conditions changed, which may be the key genes that regulate the flowering process with the change of temperature and light resources in sensitive cultivar Yongyou 538 in the late season.

## Introduction

In recent years, with continuous breakthroughs in breeding technology and the increase in residents' consumption status, the demand for high-quality rice with high yield and good flavor, especially *japonica* rice, has been rising^1^. And the area of *japonica* cultivars on the middle-lower Yangtze Plain has become an increasingly obvious trend in recent decades^2^. However, different rice varieties exhibit regionality, which is determined by the rice heading period. Determining the appropriate heading period is closely related to achieving high and stable rice yields, and it is also the key to determining the appropriate planting area and system^3^. As latitude decreases, the heading stage of rice varieties generally advances, but there are some exceptions, such as Ninggeng 4^4^, which is bred in Jiangsu Province. However, its yield is only 6.1–6.8 t ha^−1^ when planted in Zhejiang Province^5^. Therefore, analyzing the climate adaptability of *japonica* rice is of great significance for breeding and selecting *japonica* varieties with wide adaptability and high quality and yield.

Rice flowering is a complex biological process that is controlled by the genetic characteristics of the rice variety and by the external environment, including the photoperiod^6–8^, drought conditions^9–11^ and temperature^12–14^. The photoperiod pathway is considered to be the key pathway that regulates rice flowering^15^. Many of the genes involved in the photoperiod pathway in rice flowering have been successfully cloned by QTL mapping methods, such as *Heading date 1* (*Hd1*), *Heading date*-*3a* (*Hd3a*), *Early heading date 1* (*Ehd1*), *Rice Indeterminate 1* (*RID1*), etc. In addition, map-based cloning has revealed that the rice photoperiod pathway is regulated mainly by two independent signal pathways, the *OsGI*-*Hd1*-*Hd3a* pathway^7^, which is conserved with *Arabidopsis*, and unique genes such as *Ehd1*^16^, *Ehd2*^17^, *Ehd3*^18^, *Ghd7*^19^, etc. However, the functions and regulatory pathways of these genes are not fully understood. Previous studies have suggested that *Hd3a* is highly conserved among rice cultivars, and functional *RFT1* exists in most *japonica* and *indica* cultivars grown at high latitudes, while nonfunctional *RFT1* can be detected in some *indica* rice varieties grown at lower latitudes^20,21^. In addition, Kim et al.^22^ pointed out that the functional alleles of *Hd1* were present in most temperate *japonica* rice varieties, while all 12 tropical-adapted *japonica* varieties had nonfunctional *hd1* alleles, therefore, *Hd1* was considered a possible target gene for regulating flowering stage responses in different regions.

The molecular mechanisms of plant flowering have consistently been a global research hotspot. In recent years, scientists have discovered that many genes influence the rice flowering stage^23–25^. Moreover, breeders have developed a large number of high-quality and high-yielding varieties. However, few connections have been made between flowering genes and high-yielding varieties. As the most widely used sequencing technology in genomics research, transcriptome sequencing (RNA-seq) technology can be used to quickly and comprehensively obtain almost all transcript and sequence information for a specific tissue or organ of a species in a specific state. Therefore, in this study, the widely planted cultivars Ninggeng 4 and Yongyou 538 were planted on five sowing dates and two sowing dates were selected as materials for transcriptome analysis. The main objectives of this study were to (1) analyze *japonica* flowering adaptation in response to climate conditions change and (2) select the key genes regulating flowering adaptability to facilitate the spread of *japonica* rice to a wider planting area.

## Results

### Differences in yield and biomass of *japonica* cultivars among sowing dates

There was significant interaction effect of sowing date and year for the yield (Table 1). With the postponed sowing date, the yield of Ninggeng 4 remained unchanged or slightly increased, and the yield changed 9.5% (2019) and 7.2% (2020) among the five sowing dates. However, Yongyou 538 showed a trend of rising first and then decreasing or decreasing all the time. This indicated that the yield of Yongyou 538 was more sensitive to sowing date, while Ningjing 4 was relatively slow.

**Table 1:** Difference in biomass and yields of different treatments from 2019 to 2020.

Year	Treatment	Vegetative stage (g m^−2^)		Reproductive stage (g m^−2^)		Grain filling stage (g m^−2^)		Yield (t h m^−2^)	
		Ninggeng 4	Yongyou 538	Ninggeng 4	Yongyou 538	Ninggeng 4	Yongyou 538	Ninggeng 4	Yongyou 538
2019	1	493 ± 48 a	654 ± 52 a	338 ± 23 a	1000 ± 55 a	696 ± 122 b	215 ± 101 b	8.5 ± 0.1 a	9.5 ± 0.7 b
	2	313 ± 8 c	478 ± 26 b	319 ± 31 a	909 ± 73 a	745 ± 44 ab	347 ± 198 b	9.3 ± 0.6 a	10.1 ± 0.5 b
	3	465 ± 36 ab	507 ± 19 b	25 ± 35 c	248 ± 48 c	929 ± 101 a	943 ± 121 a	7.2 ± 0.4 b	9.5 ± 0.4 b
	4	439 ± 28 ab	478 ± 15 b	249 ± 60 ab	463 ± 15 b	654 ± 64 b	915 ± 23 a	8.5 ± 0.5 a	11.6 ± 0.3 a
	5	394 ± 18 bc	395 ± 19 c	217 ± 22 b	516 ± 69 b	790 ± 55 ab	801 ± 35 a	9.1 ± 0.5 a	10.0 ± 0.6 b
2020	1	377 ± 3 ab	553 ± 26 a	452 ± 29 b	564 ± 41 ab	454 ± 91 b	519 ± 54 a	6.7 ± 0.2 ab	9.3 ± 0.4 a
	2	400 ± 14 a	509 ± 35 a	266 ± 39 c	447 ± 84 b	857 ± 88 a	666 ± 155 a	6.1 ± 0.1 b	8.1 ± 0.4 b
	3	351 ± 17 b	428 ± 18 b	482 ± 14 b	629 ± 49 a	359 ± 76 b	596 ± 101 a	6.1 ± 0.3 b	8.7 ± 0.5 ab
	4	343 ± 28 b	398 ± 16 b	331 ± 31 c	577 ± 69 ab	588 ± 118 b	698 ± 86 a	7.2 ± 0.6 a	8.5 ± 0.0 ab
	5	168 ± 9 c	365 ± 16 b	602 ± 26 a	597 ± 39 ab	536 ± 54 b	572 ± 68 a	6.9 ± 0.4 ab	8.0 ± 0.0 b
**ANOVA**									
Year (Y)		107.6***	28.8***	264.0***	9.4**	42.9***	0.8 ns	170.5***	110.4***
Sowing (S)		34.8***	58.2***	26.6***	33.4***	6.0**	18.1***	10.3***	6.5**
Y*S		31.4***	6.0**	63.2***	61.9***	13.3***	13.3***	6.1**	10.6***

Data are mean and sd. Means followed by different letters at the same year are significantly different according to Tukey’s HSD test (*P* < 0.05).

***Means *P* < 0.001; **means 0.001 ≤ *P* < 0.01; *means 0.01 ≤ *P* < 0.05; ns means *P* ≥ 0.05.

There was significant interaction effect of sowing date and year for the biomass during the different stages, too (Table 1). The biomass of Ninggeng 4 decreased slightly at the vegetative stage, except for the sowing date 2 in 2019 and the sowing date 5 in 2020, and the coefficient of variation was 9.1% and 7.0% in 2019 and 2020, respectively. While Yongyou 538 showed a decreasing trend, and the coefficients of variation was 18.8% and 17.4% in 2019 and 2020, respectively. During the reproductive and grain filling period, the biomass of Ninggeng 4 and Yongyou 538 had no obvious trend among the five sowing dates, and the coefficients of variation was 39.0% and 31.4% at the reproductive stage, 25.2% and 32.2% at the grain filling stage for Ninggeng 4 and Yongyou 538, respectively. It indicated that the biomass was affected by the sowing dates, and the biomass of Yongyou 538 had a significantly higher variation range than that of Ninggeng 4.

### Differences in growth stage of *japonica* cultivars among sowing dates

In general, with the postponed sowing dates, the vegetative stage of Ninggeng 4 and Yongyou 538 was shortened, while the grain filling period was extended, and the reproductive stage was slightly different (Table 2). Compared sowing date 5 with sowing date 1, the vegetative stage of Ninggeng 4 was shortened by 3 days and 9 days in 2019 and 2020, respectively, and the effective accumulated temperature was changed by 45 °C and 47 °C in 2019 and 2020, respectively. While Yongyou 538 was shortened by 14 days and 15 days in 2019 and 2020, respectively, and the effective accumulated temperature was reduced by 173 °C and 166 °C in 2019 and 2020, respectively. The results showed that the response of different *japonica* cultivars to sowing dates was significantly different, and the difference in the vegetative stage of Ninggeng 4 was significantly lower than that of Yongyou 538, which indicated that Ninggeng 4 was insensitive to temperature and light, while Yongyou 538 was sensitive.

**Table 2:** Difference in growth period (effective accumulated temperature) of different treatments from 2019 to 2020.

Year	Treatment	Vegetative stage d (°C)		Reproductive stage d (°C)		Grain filling stage d (°C)		Growth period d (°C)	
		Ninggeng 4	Yongyou 538	Ninggeng 4	Yongyou 538	Ninggeng 4	Yongyou 538	Ninggeng 4	Yongyou 538
2019	1	51 (890)	64 (1150)	20 (395)	24 (408)	35 (556)	49 (594)	106 (1841)	137 (2153)
	2	49 (883)	59 (1080)	19 (374)	25 (427)	43 (641)	52 (575)	111 (1899)	136 (2082)
	3	49 (918)	53 (998)	21 (367)	26 (446)	39 (581)	54 (557)	109 (1866)	133 (2001)
	4	48 (911)	55 (1046)	20 (338)	20 (329)	40 (563)	58 (557)	108 (1811)	133 (1932)
	5	48 (935)	50 (977)	17 (273)	23 (365)	43 (557)	59 (514)	108 (1765)	132 (1856)
2020	1	51 (850)	61 (1059)	24 (522)	27 (555)	43 (641)	53 (597)	118 (2014)	141 (2211)
	2	49 (826)	59 (1039)	23 (498)	25 (498)	45 (622)	54 (577)	117 (1946)	138 (2114)
	3	47 (826)	54 (983)	22 (464)	25 (487)	48 (613)	55 (558)	117 (1903)	134 (2028)
	4	45 (821)	50 (937)	22 (219)	25 (461)	49 (563)	59 (564)	116 (1829)	134 (1962)
	5	42 (803)	46 (893)	24 (454)	29 (472)	51 (528)	56 (512)	117 (1786)	131 (1876)

### Correlation analysis

Table 3 showed the correlation analysis between the yield and the temperature and light resources (effective accumulated temperature, accumulated ligth hours and solar radiation) of Ninggeng 4 and Yongyou 538 during the vegetative stage from 2019 to 2020. The results showed that the yield of Ninggeng 4 was significantly positively correlated with the effective accumulated temperature and light hours at this stage, and the correlation coefficients were 0.7 and 0.8, respectively. While Yongyou 538 was significantly positively correlated with the accumulated light hours at this stage, and the correlation coefficient was 0.7. This indicated that the stable yield of Ninggeng 4 was due to its insensitivity to the vegetative stage, while the yield of Yongyou 538 decreased with the shorted vegetative period.

**Table 3:** Relation between yield and temperature and light resources in vegetative stage of different treatments from 2019 to 2020.

Variety	Effective accumulated temperature	Accumulated ligth hours	Accumulated solar radiation
Ninggeng 4	0.7*	0.8**	0.6 ns
Yongyou 538	0.5 ns	0.7*	0.4 ns

### Sequencing quality analysis of *japonica* rice tissue

The clean reads from the leaves and panicles of the *japonica* cultivars Ninggeng 4 and Yongyou 538 sown on the two sowing dates (CK vs. T) are presented in Table 4 and Table S1. In the leaves, the number of clean reads from Ninggeng 4 in the T treatment was the highest and was 0.43%, 12.85% and 16.37% higher than those from Ninggeng 4 in the CK treatment and Yongyou 538 in the CK and T treatment, respectively. In addition, in the panicles, the number of clean reads from Ninggeng 4 in the T treatment was the highest and was 4.76%, 8.68% and 2.11% higher than those from Ninggeng 4 and Yongyou 538 in the CK treatment and Yongyou 538 in the T treatment, respectively. The number of bases with a mass greater than 20 or 30 in the leaves and panicles accounted for more than 95% of the total number of bases, and the clean reads mapped to the reference genome (*Nipponbare*) were above 94% as well, indicating that the quality of RNA-seq was sufficient for the subsequent analysis.

**Table 4:** Comparison of the sequencing data for the two *japonica* cultivars sown on two sowing dates with the reference genome.

Sample	Cultivar	Treatment	Clean reads	Q20 (%)	Q30 (%)	Total mapped
Leaf	Ninggeng 4	CK	52,871,935	98.92	96.34	51,199,106 (96.84%)
		T	53,099,148	98.95	96.44	51,340,507 (96.69%)
	Yongyou 538	CK	47,050,997	98.95	96.49	45,200,802 (96.07%)
		T	45,630,931	98.50	95.36	43,236,611 (94.75%)
Panicle	Ninggeng 4	CK	49,277,341	98.84	96.14	46,713,541 (94.80%)
		T	51,622,847	98.89	96.27	49,835,160 (96.54%)
	Yongyou 538	CK	47,498,167	98.91	96.35	44,736,859 (94.19%)
		T	50,557,107	98.92	96.39	48,051,996 (95.04%)

Total reads: The number of sequences after filtering (clean reads); Q20, Q30: The percentage of bases with quality greater than 20 or 30; Total mapped: Number of clean reads that can be mapped to the genome.

### Differentially expressed genes (DEGs) under the two sowing dates

**Figure 1:**
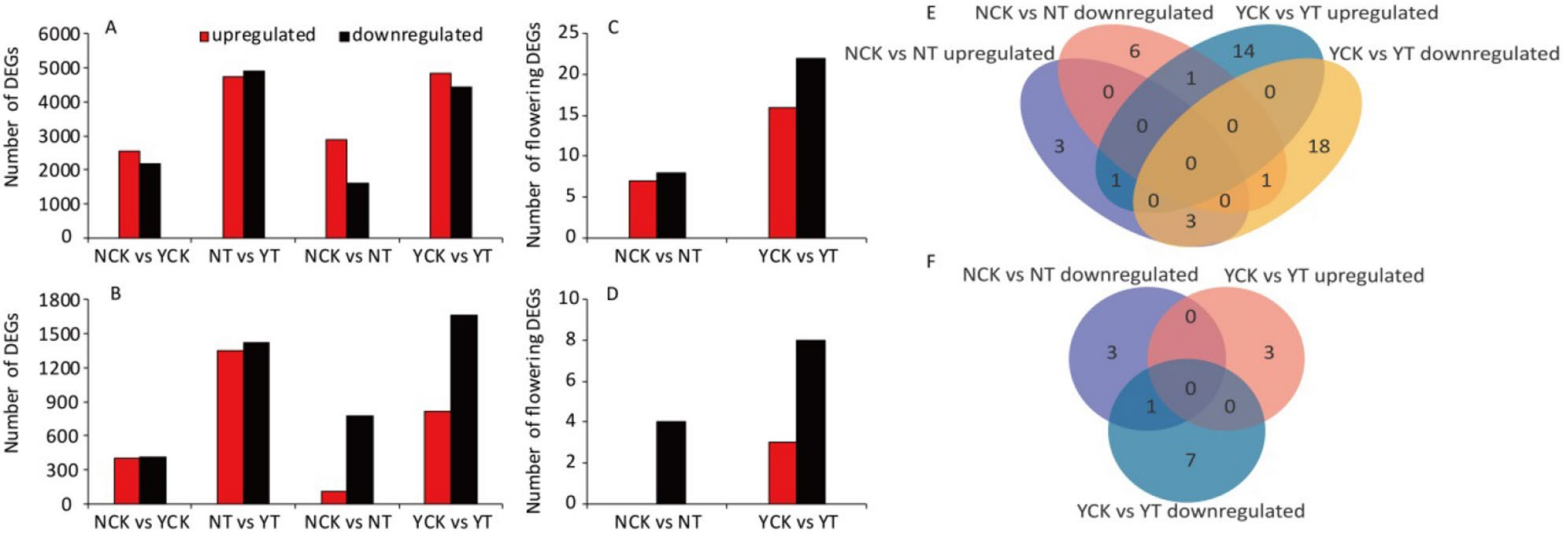
DEGs in the leaves (**A**) and panicles (**B**); flowering DEGs in the leaves (**C**) and panicles (**D**); and Venn diagrams of the DEGs in the leaves (**E**) and panicles (**F**) among the pairwise comparisons. This figure was created using Microsoft Excel 2010 and Adobe Illustraor CS6.

The two treatments in the leaves and panicles were compared to identify the DEGs between cultivars [Ninggeng 4, CK (NCK) vs. Yongyou 538, CK (YCK); Ninggeng 4, T (NT) vs. Yongyou 538, T (YT)] and sowing dates (NCK vs. NT, YCK vs. YT) (Fig. 1A,B). Comparing the NCK and YCK libraries revealed 4747 and 814 DEGs in the leaves and panicles, respectively, including 53.9% upregulated and 46.1% downregulated genes in the leaves and 48.9% upregulated and 51.1% downregulated genes in the panicles. Comparing NT and YT revealed 9622 and 2775 DEGs in the leaves and panicles, respectively, including 49.2% upregulated and 50.8% downregulated genes in the leaves and 48.8% upregulated and 51.2% downregulated genes in the panicles. Comparing NCK with NT revealed 4480 and 890 DEGs in the leaves and panicles, respectively, of which 64.2% and 12.8% were upregulated genes and 35.8% and 87.2% were downregulated genes, respectively. Comparing YCK with YT revealed 9275 and 2475 DEGs in the leaves and panicles, respectively, of which 52.3% and 32.8% were upregulated genes and 47.7% and 67.2% were downregulated genes, respectively.

The results of a functional query with the keyword “flowering” for the DEGs showed that when the sowing date was delayed 23 days, there were 7 upregulated and 8 downregulated flowering DEGs in the leaves and 4 downregulated flowering DEGs in the panicles of Ninggeng 4. In Yongyou 538, there were 16 upregulated and 22 downregulated flowering DEGs in the leaves and 3 upregulated and 8 downregulated flowering genes in the panicles (Fig. 1C,D). The Venn diagram of the flowering DEGs indicated that 6 and 1 flowering DEGs were found in the leaves and panicles of the two japonica cultivars, respectively; 9 and 3 flowering DEGs were found only in the leaves and panicles of Ninggeng 4, respectively; and 32 and 10 flowering DEGs were found only in the leaves and panicles of Yongyou 538, respectively (Fig. 1E,F).

### Analysis of flowering DEGs by KEGG pathway

**Figure 2:**
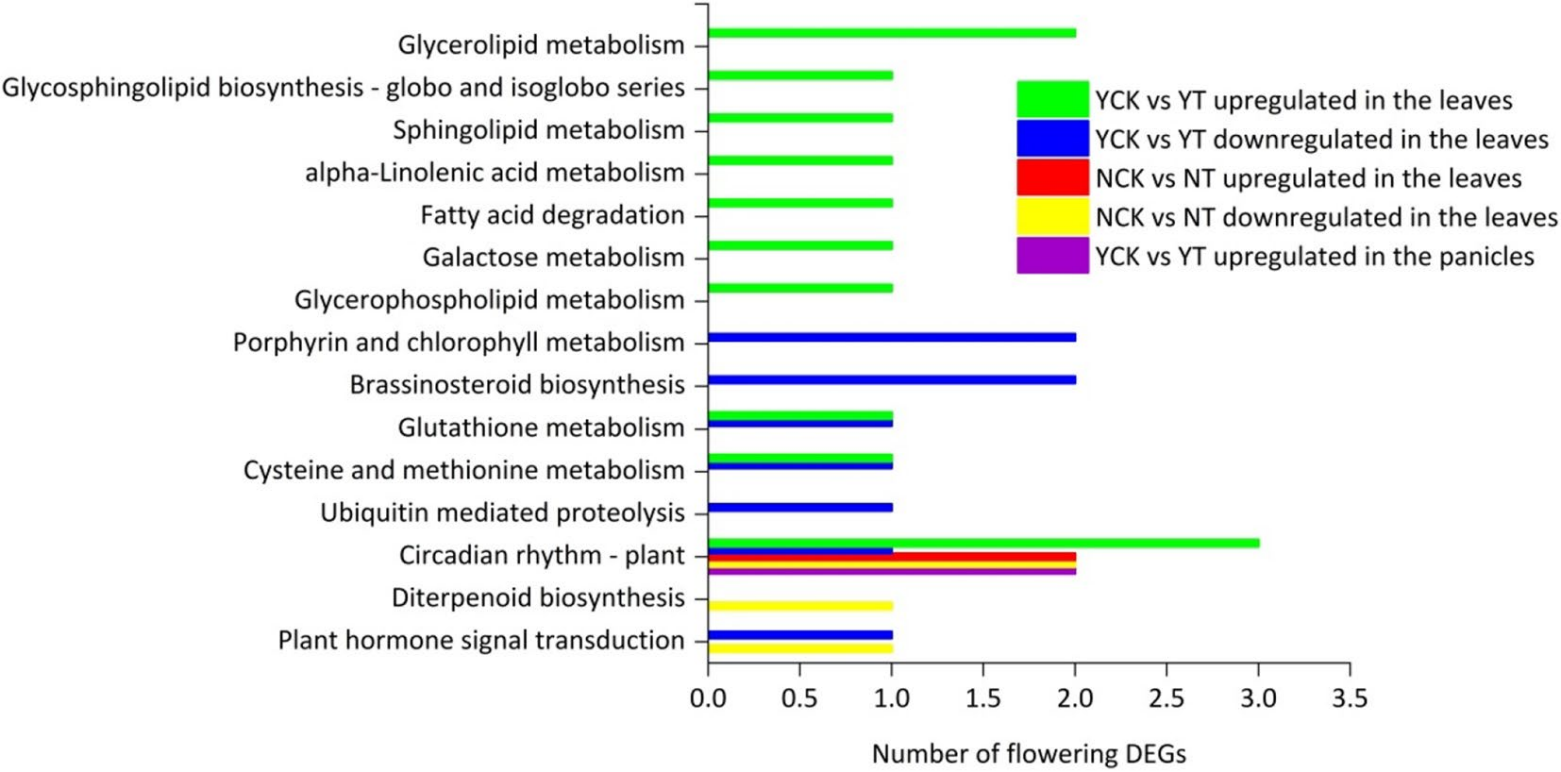
KEGG pathways of the flowering DEGs in the leaves and panicles of Ninggeng 4 and Yongyou 538 in response to different sowing dates. This figure was created using Origin 2021.

KEGG pathway analysis was carried out to further analyze the function of flowering DEGs in the leaves and panicles of Ninggeng 4 and Yongyou 538 sown on the two sowing dates (Fig. 2). In Ninggeng 4, a total of 6 flowering DEGs (2 upregulated genes *Os11g0547000* and *Os06g0552900*, 4 downregulated genes *Os09g0513500*, *Os06g0157500*, *Os03g0856700* and *Os10g0463400*) in the leaves from NCK vs NT were assigned to 3 KEGG pathways, including the plant circadian rhythm, diterpenoid biosynthesis and plant hormone signal transduction pathways, but no pathway was detected for the flowering DEGs in the panicles. On the other hand, in Yongyou 538, a total of 22 flowering DEGs (13 upregulated and 9 downregulated) in the leaves under YCK vs YT were assigned to 14 KEGG pathways, while only 2 upregulated genes (*Os01g0182600* and *Os11g0547000*)in the panicles were assigned to the plant circadian rhythm pathway. Compared with the plant circadian rhythm pathway mapped in the panicles, the upregulated genes in the leaves were significantly enriched in the plant circadian rhythm, glycerolipid metabolism, glycosphingolipid biosynthesis-globin and isoglobin series pathways, while the downregulated genes were involved in porphyrin and chlorophyll metabolism, brassinosteroid biosynthesis, glutathione metabolism, cysteine ​​and methionine metabolism, plant hormone signal transduction, plant circadian rhythm and ubiquitin mediated proteolysis pathways; of these, the porphyrin and chlorophyll metabolism pathways were significantly enriched.

### DEGs in the plant circadian rhythm pathway

**Figure 3:**
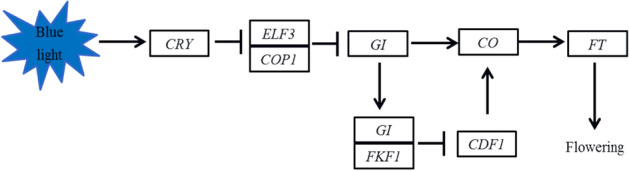
Schematic diagram of part of plant circadian rhythm pathway (map04712)^26^. This figure was created using Microsoft PowerPoint 2010.

**Figure 4:**
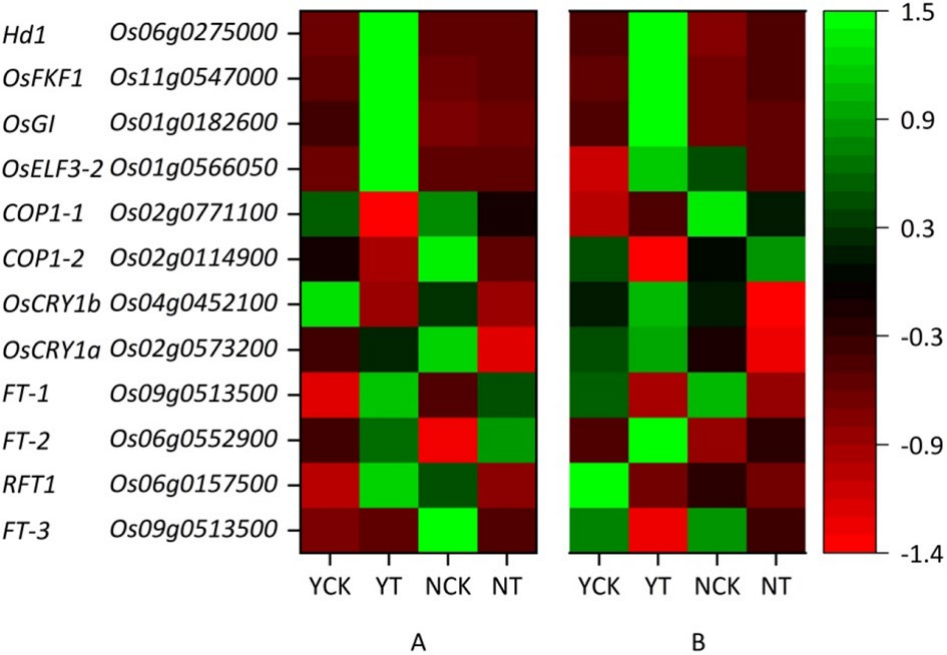
DEGs in the plant circadian rhythm pathway in the leaves (**A**) and panicles (**B**) of Ninggeng 4 and Yongyou 538 sown on two sowing dates. This figure was created using Origin 2021.

The plant circadian rhythm pathway and the DEGs under the CK vs T in the leaves and panicles of Ninggeng 4 and Yongyou 538 are listed in Figs. 3 and 4 and Table S2. The results showed that a total of 12 DEGs were involved. In the leaves, *CRY* and *COP1* were significantly downregulated, while *FKF1* and *FT* were significantly upregulated in both Ninggeng 4 and Yongyou 538, and the DEGs *Os09g0513500* (*FT-3*) and *Os06g0157500* (*RFT1*) were both downregulated in Ninggeng 4. In addition, *Os01g0566050* (*OsELF3-2*), *Os01g0182600* (*OsGI*), and *Os06g0275000* (*Hd1*) were significantly upregulated in Yongyou 538. In the panicles, only the downregulated DEGs *Os02g0114900* (*COP1-2*) and upregulated DEGs *Os01g0182600* (*OsGI*) and *Os11g0547000* (*OsFKF1*) were found in Yongyou 538.

### DEGs in the plant hormone signal transduction pathway

Among the pathways associated with the flowering DEGs under the CK vs T, the plant hormone signal transduction pathway was enriched in both Ninggeng 4 and Yongyou 538. As shown in Table 5, there were 61 and 97 DEGs in the leaves in Ninggeng 4 and Yongyou 538, respectively, and the IAA pathway had the most DEGs in both cultivars, accounting for 24.6% and 24.7% of the total number of DEGs, respectively, in which the reported genes *Os01g0741900* (*OsIAA6*) and *Os01g0764800* (*OsGH3-2*) were differentially expressed both in Ninggeng 4 and Yongyou 538. On the other hand, we found that two flowering DEGs were significantly downregulated in the CTK pathway, including *Os10g0463400* (*B-ARR*) in Ninggeng 4 and *Os10g0362300* (*CRE1*) in Yongyou 538. In addition, *Os01g0718300* (*OsBRI1*), which is involved in the BR pathway, was significantly downregulated in Yongyou 538. Although there were 19 and 35 DEGs in the panicles of Ninggeng 4 and Yongyou 538, respectively, no flowering DEGs were found in the panicles.

**Table 5:** DEGs in the plant hormone signal transduction pathway in the leaves and panicles of Ninggeng 4 and Yongyou 538.

Plant hormone	NCK versus NT				YCK versus YT			
	Leaf		Panicle		Leaf		Panicle	
	Upregulated	Downregulated	Upregulated	Downregulated	Upregulated	Downregulated	Upregulated	Downregulated
IAA	4	11	0	1	12	12	1	5
CTK	1	6	0	3	2	9	1	2
GA	0	4	0	1	1	5	0	1
ABA	6	2	0	1	13	7	3	7
ETH	2	7	0	2	4	9	1	2
BR	1	2	0	0	3	4	1	0
JA	7	1	0	9	0	11	0	8
SA	4	3	0	2	3	2	1	2

IAA: Auxin; CTK: Cytokinin; GA: Gibberellin; ABA: Abscisic acid; ETH: Ethylene; BR: Brassinosteroid; JA: Jasmonic acid; SA: Salicylic acid.

### qRT-PCR for DEGs

**Figure 5:**
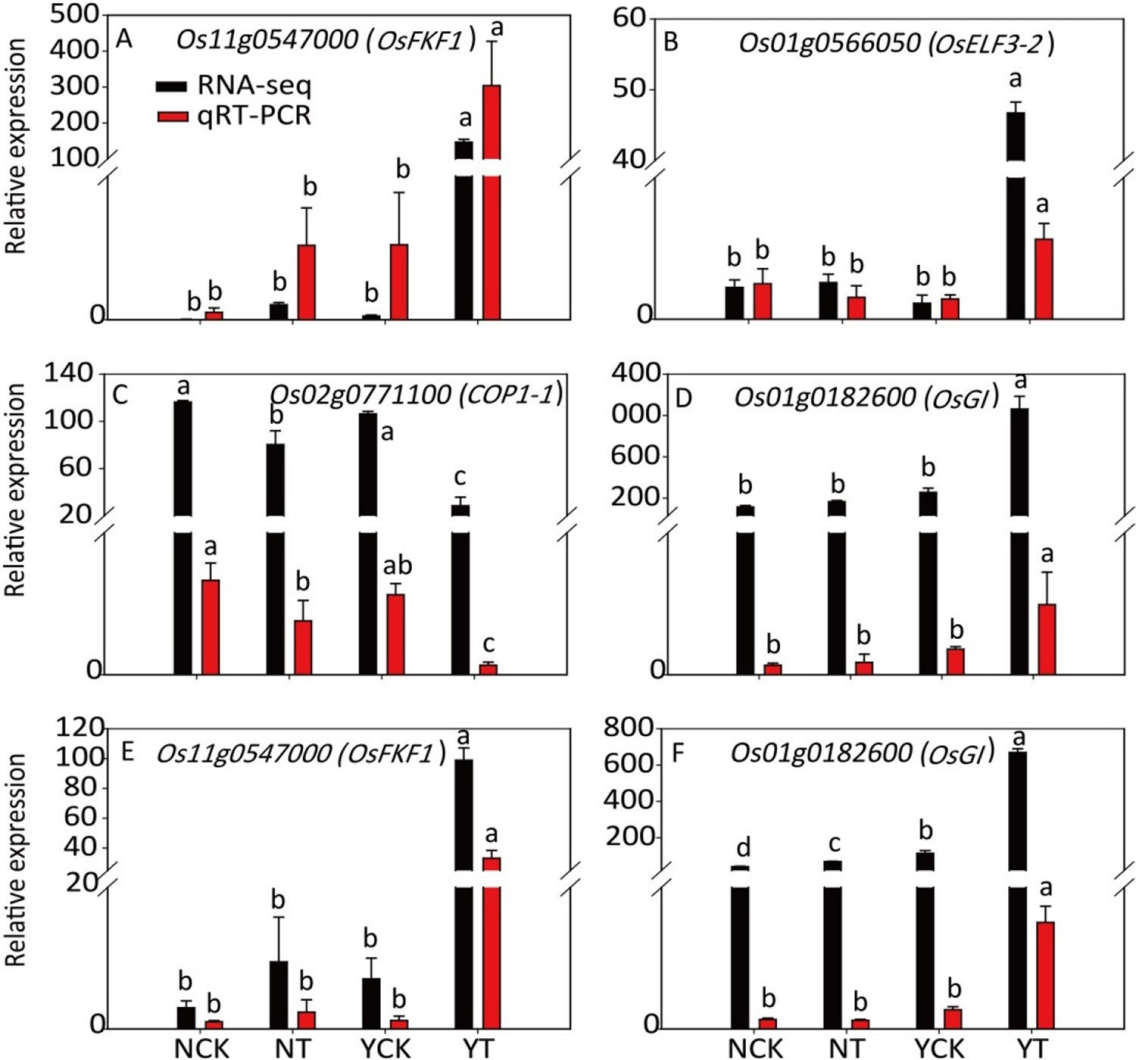
The relative expression of DEGs in the leaves (**A**–**D**) and panicles (**E**,**F**) of Ninggeng 4 and Yongyou 538 sown on two sowing dates. This figure was created using Origin 2021 and Adobe Illustraor CS6. Values are the means and sds. Means followed by different letters in the same test method are significantly different according to Tukey’s HSD test (*P* < 0.05).

Four DEGs involved in the plant circadian rhythm pathway were analyzed by qRT-PCR. The results showed that the expression patterns detected in the qRT-PCR were similar to those detected with RNA-seq (Fig. 5), indicating that the RNA-seq data used in this experiment were reliable.

## Discussion

**Figure 6:**
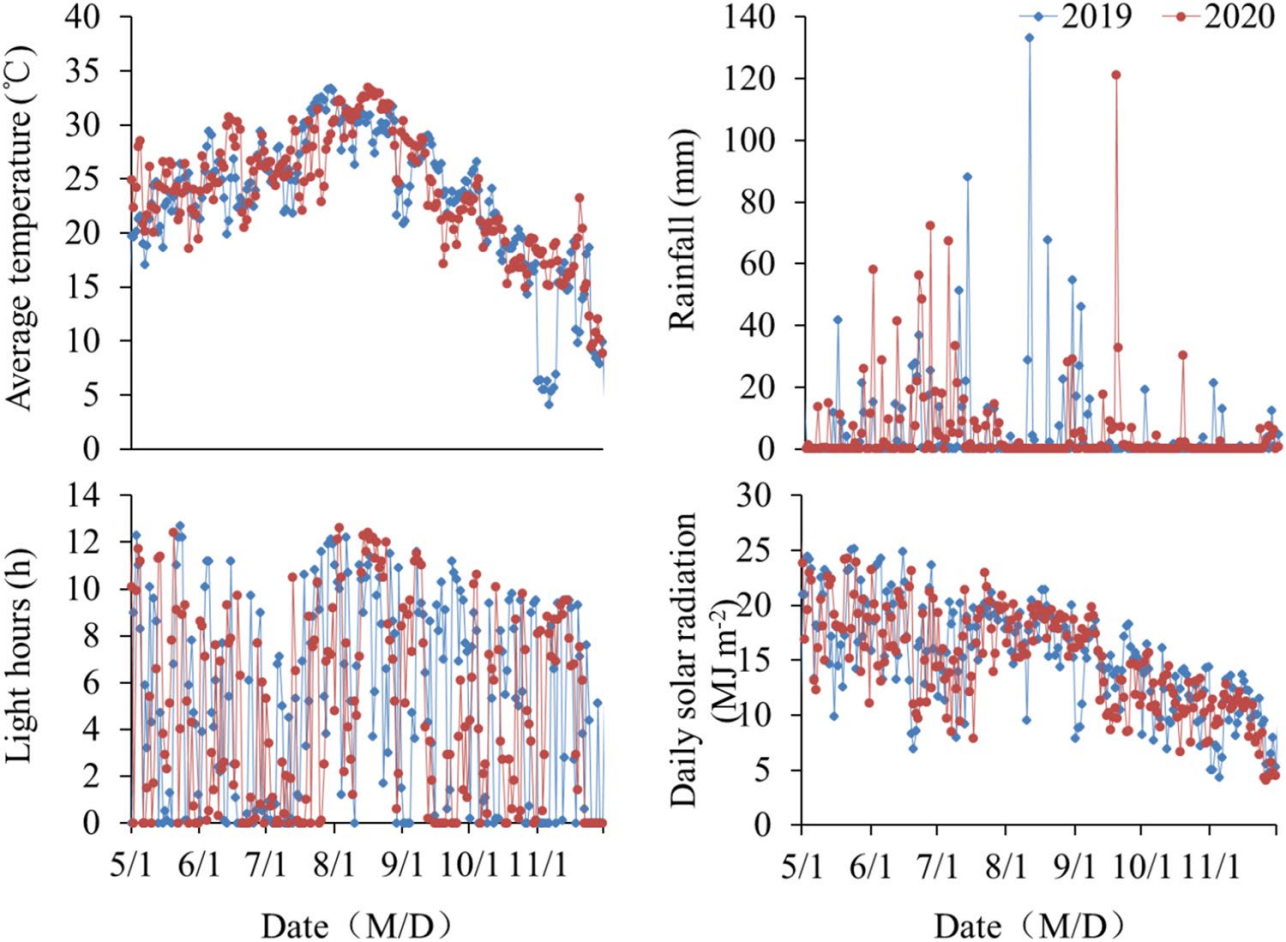
Changes in the climate resources from 2019 to 2020. This figure was created using Microsoft Excel 2010.

In our study, when the sowing date was delayed by 23 days, the days-to-heading of Ninggeng 4 and Yongyou 538 were occurred 6 and 15 days earlier than that in the sowing date 1 (CK), respectively. In the different growth stages, the vegetative and reproductive periods of Ninggeng 4 were both decreased by 3 days, while the vegetative and reproductive stages of Yongyou 538 were decreased by 14 and 1 day, respectively (Table 2). This means that the change in the days-to-heading occurred mainly in the vegetative growth period, which is consistent with the results of a previous study^27,28^. During the vegetative stage, the average daily temperatures for Ninggeng 4 and Yongyou 538 under the T treatment were 29.5 °C, which was 7.4% and 5.6% higher than those in the CK treatment and close to the optimum temperature for *japonica* growth and development (30 °C) (Fig. 6). Duan et al.^29^ pointed out that the growth rate of *japonica* was accelerated and its growth stage was shortened when the average daily temperature increased during the vegetative growth period and that *japonica* showed different degrees of temperature sensitivity. Similarly, the sensitivity of the responses of Ninggeng 4 and Yongyou 538 to temperature and light changes were not consistent, and Yongyou 538 was more sensitive than Ninggeng 4. The greater number of DEGs in Yongyou 538 than in Ninggeng 4 also supports this idea (Fig. 1A–D). In addition, more DEGs were detected in the leaves than in the panicles (Fig. 1A–D), indicating that leaves, rather than panicles, are the organs that perceive climate conditions change. This finding is similar to that in a previous study showing that plants perceive the change in light hours with their leaves and initiate stem apex growth and flower formation based on these perceptions^30^.

The biological clock of plants plays an important role in regulating their flowering process. Under different photoperiod treatments, the plant flowering stages can be affected by the genes involved in the plant circadian rhythm pathway. In our study, as the climate conditions changed, DEGs under CK versus T in the circadian rhythm pathways were observed in both Ninggeng 4 and Yongyou 538 (Fig. 2). The meteorological data during this period showed that the average daily light hours decreased by 25.4% and 6.3% and the accumulated light hours decreased by 36.6% and 10.2% from panicle initiation to heading in Ninggeng 4 and Yongyou 538, respectively (Fig. 6). A previous study showed that when the blue light receptor CRY in plants receives blue light signals, the activity of its downstream gene *COP1* is inhibited^31^. As a result, *COP1* was significantly downregulated both in Ninggeng 4 and Yongyou 538 in the sowing date 5 (T) compared with sowing date 1 (CK) (Fig. 4). *COP1* can interact with its downstream gene *ELF3* and regulate the biological activity of light signals input by the downstream target *GI* to regulate the circadian rhythm pathway and the flowering stage^32^. In addition, *OsELF3-2* was significantly upregulated in Yongyou 538 (Fig. 3). Yu et al.^32^ pointed out that the function of ELF3 in regulating plant circadian rhythms required complete COP1 activity. Therefore, the downregulation of *COP1* in Yongyou 538 resulted in increased expression of *GI* (Fig. 4). As a gate switch for light signal inputs, the homologous gene of *GI* in *Oryza sativa*, *OsGI*, activates the transcription of *Hd1* (the homologous gene of *CO* in *Oryza sativa*) under short-day light conditions^7^. In addition, GI can combine with FKF1 to form a blue-light-dependent protein complex, which can indirectly promote the transcription of *CO* by degrading CDFs (the transcription repressors for *CO*)^33–35^. Subsequently, *CO* can promote the differentiation of flower primordia to form flower tissues by inducing the transcription of *FT*^36^. Therefore, *OsFKF1*, *Hd1* and *FT-1* were upregulated with the upregulation of the upstream gene *GI* in Yongyou 538 in our study (Fig. 3). In Ninggeng 4, *OsFKF1* and *FT*, including *FT-2*, *FT-3* and *RFT1*, expressed differentially compared CK and T, while *OsGI* was not differentially expressed (Fig. 4), resulting in a small change in the number of protein complexes formed by FKF1 and GI. As a result, the interaction of upregulated genes and downregulated genes in *FT* may be the main reason for the difference in the flowering stage of Ninggeng 4 under the two sowing dates.

In addition to their biological clocks, plants can also regulate the flowering process with hormones. Our research found that *B-ARR* was significantly downregulated in Ninggeng 4 through the CTK transduction pathway. Previous studies have shown that cytokinins can regulate the rice flowering process through genes such as *AHK2* and *AHK3*^37^, *B-ARR* and *A-ARR*^38^. In *Oryza sativa*, *Ehd1* is highly homologous to *B-ARR* and can interact with the *A-ARR* protein OsRR1 to form a heterodimer and inhibit the activity of Ehd1^38^. In terms of photoperiod regulation, *Ehd1* can directly regulate its downstream gene *FT* and promote the flowering stage under short-day light conditions^16^. Therefore, two downregulated genes in *FT* were detected in Ninggeng 4 (Fig. 4). In addition, *BRI1* was significantly downregulated in Yongyou 538 through the BR transduction pathway. In this pathway, *BRI1* is a receptor gene on the membrane and can promote dark morphogenesis and early flowering^39^. However, the effect of *BRI1* in Yongyou 538 may have been weakened by other flowering genes in our study. In addition, DEGs also existed in the signal transduction pathways of IAA, GA, ABA, Eth, JA, and SA (Table 5), which have been confirmed to play a role in rice flowering^40–44^. The number of hormone-related DEGs, especially those related to IAA, was higher in the sensitive cultivar Yongyou 538 than in the insensitive cultivar Ninggeng 4. This result indicates that hormones play an important role in *japonica* flowering in response to climate conditions change.

Two different subspecies of rice were selected as the materials for this research. A previous study showed that the functional diversity of flowering-time gene sequences has no special relationship between rice subspecies^45^. Gene expression analysis showed that the expression of *Os01g0566050* (*OsELF3-2*), *Os11g0547000* (*OsFKF1*), *Os01g0182600* (*OsGI*), *Os06g0275000* (*Hd1*) and *Os09g0513500* (*FT-1*) was slightly or significantly higher in the sensitive cultivar Yongyou 538 after the sowing date delay (YT) than other treatments (NCK, NT and YCK), while the expression of *Os02g0771100* (*COP1-1*) was significantly lower, these may be the key genes that regulate the flowering process of *japonica* cultivars adapting to different planting areas. However, this finding needs to be verified by further experiments.

## Conclusions

The flowering stage in the two *japonica* varieties responds differently to climate change. Yongyou 538 is more sensitive and affected by the delay in the sowing date than Ninggeng 4, as indicated by its higher number of DEGs. Therefore, Yongyou 538 can adapt to climate conditions change by modulating the plant circadian rhythm and hormone signal transduction pathways. Gene expression analysis indicated that *Os01g0566050* (*OsELF3-2*), *Os01g0182600* (*OsGI*), *Os11g0547000* (*OsFKF1*), *Os06g0275000* (*Hd1*), and *Os09g0513500* (*FT-1*) were expressed higher and *Os02g0771100* (*COP1-1*) was expressed lower in the sensitive cultivar as the climate conditions changed, these may be the key genes that regulate the flowering process in *japonica* cultivars adapting to different planting areas.

## Materials and methods

### Study site description

Field experiments were carried out in 2019 and 2020 at the farm (30°05′N, 119°90′E, 21 m altitude) of the China National Rice Research Institute in Hangzhou, Zhejiang Province. The field soil properties were as follows: organic matter, 30.5 g kg^−1^, tatal N, 1.7 g kg^−1^, available P, 14.2 mg kg^−1^, available K, 140.2 mg kg^−1^, and pH, 5.8. The weather information, including the average temperature, rainfall, light hours and solar radiation from May to November of 2019 and 2020, are shown in Fig. 6.

### Experiment design

The rice varieties Ninggeng 4 (N) and Yongyou 538 (Y) were selected as the experimental materials, and five sowing dates were conducted in 2019 and 2020. Pregerminated seeds were sown in seedbeds. The sowing and transplanting date were listed in Table 6. The hill spacing was 25 cm × 16.7 cm. The total N application rate was 202.5 kg ha^−1^, 50% was applied 1 day before transplanting (N:P_2_O_5_:K_2_O 15:15:15; compound fertilizer), 30% was applied 14 days after transplanting (urea), and 20% was applied at the panicle initiation stage (urea). Approximately 100.0 kg ha^−1^ P_2_O_5_ and K_2_O in the form of compound fertilizer were applied as basal fertilizer 1 day before transplanting, and an additional 65.0 kg ha^−1^ K_2_O in the form of potassium chloride was applied as a topdressing at the panicle initiation stage.

**Table 6:** Sowing and transplanting date of different treatments from 2019 to 2020.

Year	Treatment	Sowing date	Transplanting date
2019	1	2019/6/16	2019/7/3
	2	2019/6/21	2019/7/9
	3	2019/6/27	2019/7/17
	4	2019/7/2	2019/7/23
	5	2019/7/9	2019/7/30
2020	1	2020/6/10	2020/6/29
	2	2020/6/17	2020/7/6
	3	2020/6/24	2020/7/13
	4	2020/7/1	2020/7/20
	5	2020/7/8	2020/7/27

### Measuerments

In 2019 and 2020, 6 hills were sampled from each plot at the tillering (approximately 15–20 days after transplanting), panicle initiation, heading, and maturity stage. The samples were separated into leaf, stem and panicles and then oven dried for 72 h at 75 °C. The aboveground biomass was calculated by summing the dry weight of all organs. The yield was converted to 14% moisture content. The heading stage was defined as the date when 80% of the panicles had emerged, and the plant reached maturity when 95% of the spikelets had turned yellow.

The flag leaves and panicles of Ninggeng 4 and Yongyou 538 in sowing date 1 (considered a control check, CK) and sowing date 5 (considered a treatment, T) in 2019, respectively, were removed at the flowering stage, packed in tinfoil, quickly placed in liquid nitrogen, and then stored in a − 80 °C freezer for subsequent RNA extraction. Three biological replicates of each sample were taken.

### cDNA library preparation and RNA-seq

The library was constructed using an Illumina TruSeq TM RNA Sample Prep Kit method. Total RNA was extracted from the leaves and panicles of Ninggeng 4 and Yongyou 538 from the CK and T treatments. Its concentration and purity were detected by a Nanodrop2000. The integrity of the RNA was detected by agarose gel electrophoresis, and the RNA integrity number (RIN) value was detected by Agilent 2100. Then mRNA was enriched by oligo-dT selection, and fragmentation buffer was added to fragment the mRNA. Under the action of reverse transcriptase, a random six-base primer was added, and cDNA was synthesized by reverse transcription using mRNA as a template. The cDNA was then connected to the adaptor. The library was enriched and amplified in 15 PCR cycles. The target band recovered from the 2% agarose gel was quantified by TBS380 Picogreen and mixed in the indicated ratios. Clusters were generated by bridge PCR amplification on cBot. Finally, the PE library was sequenced on the Illumina platform with a read length of 2 × 150 bp.

### Information analysis

Tables and figures were processed by Microsoft Excel 2010 and Microsoft PowerPoint 2010 ((Microsoft, Redmond, WA, USA), Origin 2021 (OriginLab, Northampton, MA, USA) or Adobe Illustraor CS6 (Adobe, San Jose, CA, USA). SAS 9.4 (SAS Institute Inc., Cary, NC, USA) was used to analysis of variance (ANOVA). Two-way ANOVA was used to compare the effects of year and sowing date with a general linear model (GLM). One-way ANOVA was performed to evaluate the effects of sowing date on biomass, yield and growth period parameters. Means were subjected to Tukey’s honestly significant difference (HSD) test at the 0.05 probability level. The raw reads obtained by Illumina sequencing were filtered to obtain high-quality sequencing data (clean reads). TopHat2 was used to compare the clean data with the *japonica* reference genome *Nipponbare* (https://www.ncbi.nlm.nih.gov/assembly/GCF_001433935.1/) for gene sequence comparison, obtain the mapped data and evaluate the quality of the sequence results. The gene expression amounts were calculated and normalized as transcripts per million (TPM). The thresholds of |log_2_FC|> = 1 and padjust < 0.05 were used to screen for differentially expressed genes (DEGs) with DESeq2. Then, the DEGs were associated with KEGG pathways, which were considered significantly enriched at P value_uncorrected < 0.05. The sequencing and preliminary analysis in this experiment were completed by Shanghai Majorbio Biopharm Technology Co., Ltd.

### Real-time fluorescence quantitative PCR (qRT-PCR) analysis

Four DEGs involved in KEGG pathways were further analyzed by qRT-PCR. A ChamQ SYBR Color Master Mix (2X) (Nanjing Novazan Biotechnology Co., Ltd.) reverse transcription kit was used in the ABI 7500 Real-Time PCR System. The internal reference gene was actin, and the relative expression of the genes was calculated by the 2^−∆ ∆Ct^ method. The primer sequences are shown in Table 7.

**Table 7:** qRT-PCR primers.

Gene	Forward primer	Reverse primer
*Os11g0547000*	5′-GATGAGCACCAAGATGTTAGG-3′	5′-CAGGCTTTGCAGATTCCAG-3′
*Os01g0566050*	5′-AACAGCGAGCATCAAGCG-3′	5′-CACCAATGGCACCAACAA-3′
*Os01g0182600*	5′-CAGCGGAAGATTACGATT-3′	5′-CGATGATACATAGCCACCT-3′
*Os02g0771100*	5′-ACTTTGTTGGGCTGTCTG-3′	5′-TAGGGCTATCGCTCTTCC-3′

### Ethics declarations

Experimental research and field studies on cultivars have complied with relevant institutional, national, and international guidelines and legislation. And all cultivars were purchased through formal channels.

## Supplementary Information

Supplementary Table S1.

Supplementary Table S2.
